# New Trends in Biotechnological Processes to Increase the Environmental Protection

**DOI:** 10.1155/2013/138018

**Published:** 2013-11-17

**Authors:** Ana Moldes, José Manuel Domínguez González, Ligia Raquel Marona Rodrigues, Attilio Converti

**Affiliations:** ^1^Chemical Engineering Department, University of Vigo, School of Industrial Engineering (EEI), Campus As Lagoas Marcosende, Vigo, 36310 Pontevedra, Spain; ^2^Chemical Engineering Department, University of Vigo, Faculty of Sciences, As Lagoas s/n, 32004 Ourense, Spain; ^3^Institute for Biotechnology and Bioengineering (IBB), Centre of Biological Engineering, University of Minho, Campus de Gualtar, 4710-057 Braga, Portugal; ^4^Department of Civil, Chemical and Environmental Engineering, Pole of Chemical Engineering, University of Genoa, Via Opera Pia 15, 16145 Genoa, Italy

A sustainable development can be achieved by deepening into more effective and eco-friendly products and technologies. From this point of view, the development of biotechnological processes to increase the environmental protection could be included in the best available techniques reference documents, the so-called BREFs, that cover, as far as practicable, the industrial activities to achieve an integrated pollution prevention and control. Members of the European Union are required to take these documents into account when determining the best available techniques, generally or in specific cases under the European Commission Directives.

In order to include biotechnological processes into the BREFs, for example, for obtaining food and pharmaceutical additives, these products have to be cost competitive with those synthesized by chemical ways. Biotechnological processes are advantageous compared to the chemical ones since various metabolites can be obtained simultaneously in the same process, and these metabolites are more eco-friendly than their chemical counterparts. Additionally, this feature also matches the increasing demand of consumers for natural products, which has intensified the biotechnological production of natural additives.

This special issue reports advances in the use of biotechnological processes for the treatment of contaminated soil or water as well as the revalorization of agroindustrial residues through the production of valuable metabolites such as biosurfactants or antioxidants, with applications in biomedicine, food industry, pharmaceutical industry, or environmental bioremediation.

Therefore, to avoid or at least to reduce the use of synthetic additives, it is necessary to identify natural alternative sources of food and pharmaceutical additives. For instance, in the recent years, there has been a growing interest in the use of natural antioxidants in the food industry not only for application as preservatives but also due to their benefits to human health. Moreover, the brewery industry generates waste that could be used to produce natural extracts containing bioactive phenolic compounds, which can be further used as natural antioxidants with potential applications in the food, cosmetic, and pharmaceutical industries.

On the other hand, water and soil pollution has become a significant environmental issue around the world. Toxic substances, such as endocrine disruptors, heavy metals, and excessive inflows of phosphorus, nitrogen, and other elements, all contribute to the water and soil pollution. Biotechnological processes, as well as metabolites extracted from plants, can be used to treat those contaminations enabling the use of mild operating conditions, as compared with other physical and chemical treatments, thus permitting a sustainable development. Accordingly, anammox bacteria can be used for the elimination of ammonium in wastewater, whereas phosphorus can be removed from water by using transgenic plants, which hyperaccumulate inorganic phosphate.

In addition, in this special issue it has been showed that epipsammic biofilms can play an important role in the environmental quality of river systems, increasing arsenic and phosphorous retention by the system, especially in environments where both arsenic and phosphorus occur simultaneously, whereas vegetables and vegetable residues can be used as sources of enzymes capable of removing colored compounds from water.

Regarding soil bioremediation, biosurfactants produced by *Lactobacillus pentosus* were found to improve the solubilization of hydrocarbons in the water phase of soil, leading to even better results than those reached with common chemical surfactants like sodium dodecyl sulfate.

